# Traditional Meditation, Mindfulness and Psychodynamic Approach: An Integrative Perspective

**DOI:** 10.3389/fpsyg.2016.00552

**Published:** 2016-04-21

**Authors:** Simone Bianco, Paolo Barilaro, Arianna Palmieri

**Affiliations:** ^1^Department of Philosophy, Sociology, Education and Applied Psychology, University of PadovaPadova, Italy; ^2^Cognitive Neuroscience Center, University of PadovaPadova, Italy

**Keywords:** psychodynamic, meditation, mindfulness, integration, psychotherapy

## Introduction

In the last three decades, we have been witnessing a growing interest toward academic research on mindfulness practices based on Traditional Meditation (TM) and Buddhist precepts (Pagnini and Philips, 2015).

Phenomenologically, meditation is a practice that could be meant as mind focalization on objects, body feelings, emotions and thoughts. It could have religious, spiritual, and philosophical purposes, but it can just be aimed to a deeper knowledge of themselves and/or an improvement of psychophysical condition.

Under a Buddhist's perspective, causes of all human sufferings should be eradicated through a progressive recognition of the real nature of the Self as impermanent and interdependent: meditation is meant as the practice aimed to achieve this goal (Dalai Lama, 1986).

This process of liberation from suffering is based on Samatha and Vipassana practices, as two-sided aspects of TM. The former is a concentrative meditation; this is a “*practice in which sustained attention is developed by attending to the target object to the total exclusion of all other objects and experiences*” (Rapgay and Bystrisky, 2009, p. 154). The latter is an insight-based meditation; this is a practice in which awareness of impermanence and interdependence of Self and reality are obtained through observation of phenomena (Goleman, 1976).

Western psychotherapy approaches have been integrating their clinical practices with these Eastern techniques and doctrines. After the rise of Mindfulness-Based Stress Reduction program, built up by Kabat-Zinn (1990), many protocols, the so-called Mindfulness-Based Interventions (MBIs), spread out. To mention some examples among MBIs: Dialectical Behavior Therapy (DBT - Linehan, 1993), Acceptance and Commitment Therapy (ACT – Hayes et al., 1999), Mindfulness-based Cognitive Therapy (Segal et al., 2002).

MBIs are typically grounded on a cognitive-behavioral perspective (for an overview see Mace, 2007) and usually they use meditation as a method to improve “acceptance of” and “focal attention on” the immediate present moment in a nonjudgmental way (Bishop et al., 2004; Rapgay and Bystrisky, 2009). A number of randomized control trials has tested MBIs efficacy showing improvements in many medical and/or psychological disorders, such as depression and anxiety (Bohlmeijer et al., 2010; Hofmann et al., 2010; Piet and Hougaard, 2011; Piet et al., 2012; Thompson et al., 2015) and neurological conditions (Rosenzweig et al., 2010; Pagnini et al., 2015; for a review of the literature, see Grossman et al., 2004; Keng et al., 2011; Galante et al., 2012, 2014; van der Velden et al., 2015).

Recently, TM and MBIs have been compared in order to underline theoretical and practical similarities and differences. Even if MBIs have demonstrated their efficacy, it seems that relevant components of TM may have been developed in a peculiar way, leading to a modern mindfulness concept that partially diverge from traditional, ancient one (Rapgay and Bystrisky, 2009; Chiesa and Malinowski, 2011). For examples, at a theoretical level: TM and MBIs are similar since they both refer to meditation as main method to achieve their goals, and both of them assume that a mental training, to calm mind and develop self-observation skills, is required to reach psychological changes; but they are different in their purposes, since MBIs are oriented toward psychological welfare and treatment of psychopathologies (Kabat-Zinn, 1990; Segal et al., 2002), whilst TM aims at reducing humans' afflictions through a path toward the comprehension of the nature of the Self (Dalai Lama, 1986; Epstein, 2008). Practically, both TM and MBIs use Samatha and Vipassana meditation, but in MBIs they are not taught as two different practices as in TM (Shapiro and Carlson, 2009).

Moreover, some MBIs, such as the DBT or the ACT, seem to be mostly content-focused: they are oriented to a modification of cognitions, thoughts and emotions (Chiesa and Malinowski, 2011). Conversely, TM is mostly process-focused and it is mainly concerned with introspective awareness of pure perceptions (Rapgay and Bystrisky, 2009).

In synthesis, meditation practices have been efficaciously integrated in Western cognitive-behavior-oriented psychology, but some features of TM had been changed or not considered in the modern concepts of mindfulness (to deepen, see Rapgay and Bystrisky, 2009; Chiesa and Malinowski, 2011).

## Theoretical background

Psychodynamic interventions are one of the most diffuse psychological approach in Western countries. The basic assumption of psychodynamic perspective is that thoughts and behaviors are determined by unconscious and often conflictual dynamics out of awareness. Ego functions, object relations relevance, defense mechanisms, intra-psychic conflict, transference and countertransference phenomena, and the relevance of past events on personality are some of the main theoretical concepts of these approaches (Fonagy and Target, 2009; Gabbard, 2014). Purposes of psychodynamic psychotherapy are various, partially depending on specific clinical orientation, but generally, they are focused toward the intra-psychic conflict resolution and the research of the truth about the Self, bringing unconscious material to awareness.

In our opinion, it is very peculiar that TM has not been well integrated into psychodynamic approach. These perspectives share many concepts and features: they are both introspective disciplines that aim to help humans in coping with psychological suffering, to find the truth about the self, and to assist awareness of unconscious processes in order to make people “*psychologically free*” (Germer et al., 2013).

We carried out a research on PubMed using the key words “psychodynamic and mindfulness,” “psychodynamic and meditation,” “psychodynamic and Buddhism,” from 1990. On the basis of this simple search, we found out 24 articles. For the most, these papers were focused on the comparison between different psychotherapy models, such as psychodynamic psychotherapy and MBIs (Sørensen et al., 2011; Flynn, 2012; Hunot et al., 2013; Mayo-Wilson et al., 2014; Bandelow et al., 2015; van Dessel et al., 2015) and furnish a review of the different models applied in specific situations, such as sexual dysfunction, chronical pain, irritable bowel syndrome (Naliboff et al., 2008; Assalian, 2013; Purdy, 2013; Recordon and Köhl, 2014).

Actually, this scientific literature seems to highlight the differences and the peculiarities of these perspectives rather than the overlapping features and the possibilities to integrate.

Nevertheless, there are a few attempts to integrate meditation principles and psychodynamic approaches: Johansson et al. (2012) applied mindfulness measures to evaluate the process of an “online” psychodynamic psychotherapy; Schussel and Miller (2013) used a method called “best self visualization,” which is aimed at increasing self-efficacy and reintegrating dissociated self-states, and they described it within the framework of meditation and psychodynamic theory; Twemlow (2001a,b) proposed to use specific precepts of Zen's practice to train psychodynamic therapists.

The only integrative structured model we found is the Mindfulness-Based Transactional Analysis (Žvelc et al., 2011), in which modern mindfulness techniques have been combined to Transactional Analysis, that is a psychodynamic-oriented approach at theoretical level. However, there is not yet any empirical study aimed to evaluate its efficacy.

To the best of our knowledge, scientific literature completely lacks of any integrative RCT involving the use of meditation in the psychodynamic clinical context.

However, Eastern conceptions of human psyche in Western psychology tradition has been already introduced by preeminent figures from European psychoanalytical panorama, such as Jung (1939), Fromm et al. (1960), and more recently, scholars as Stern (1997), and Safran (2003).

For instance, Jung's perspective is focused on the individuation process aimed at obtaining self-awareness of unconsciousness. This process permits a reduction of unconscious material influence on oneself through a widening of conscience. It could be described as an experience that brings to the discovery and acceptance of the real nature of Self, and the awareness of its relationship with all the other people and creatures.

By congregating the Freudian tradition with the Jungian perspective, Fromm explicitly recommends many contact points between Psychoanalysis and Buddhism, such as the same ethical orientation, similar goals, the need of a guide (master/psychotherapist) for the practice, and the idea that intellectualization is uselessness in the quest of oneself.

Stern focused on a radical awareness of *hic et nunc*. He claims that an aware and non-judgmental attention to the present moment could further a deeper understanding of oneself and others. This feature is very similar to traditional and modern mindfulness approaches.

Moreover, the Safran's relational perspective supports the conception of a Self as an ensemble of relational configurations, as it grows up in a contextual situation. Interestingly, this idea is partially overlapping the Buddhist perspective of an interdependent and contingent Self.

## Toward an integration between psychodynamic and meditation practices

Although these few but authoritative theoretical efforts and contributions, the integration process of meditation in psychodynamic psychotherapy has a number of criticisms mainly at theoretical level. These limits could have impeded psychodynamic therapists in applying meditation-based interventions during their clinical practice.

Part of this issue could be due to the conceptual ambiguity concerning the terms “Ego,” “Self,” and “emptiness,” which makes TM and psychodynamic perspectives apparently incompatible. Indeed, on one side, concepts as “focal attention” or “awareness of the present moment” had been translated easily into the typical language of cognitive-behavior-oriented psychology; on the other side, the integration of Buddhism and psychodynamic assumptions of Ego seems to be more complex (Rubin, 1999). If Buddhism is conceived as an “egolessness” discipline and psychodynamic as an “ego-centered” approach (Hwang, 2011), it is necessarily impossible to build an inter-theoretical bridge between these two perspectives. Meditation is indeed described as a method directed to transcend Ego, whilst psychodynamic psychotherapy is classically aimed at strengthening Ego (Engler, 1984). Recently, Shiah (2016) positioned the Self (meant as pleasure-oriented) and the Nonself (meant as the realization that the Self lacks intrinsic existence) at the opposite ends of a continuum. In this framework, meditation is suggested as a method to promote the dissolution of the pleasure-oriented Self, and the acquisition of insights about the real nature of all things, toward a Nonself state. This represents an interesting translation of Buddhist psychology in a Western language, but Self-transcending processes still need further explorations for a better understanding of their dynamics.

In fact, conversely to common-sense language, during meditation some aspects of Ego, as conceived by Western psychological perspective, are boosted, and *not* abandoned. Meditation influences many positive aspects of Ego, enabling a development from *inside*, and not *beyond* Ego itself (Epstein, 2008). By considering Ego like a combination of functions and representations (Epstein, 1988, 2008), it is possible to understand in a Western perspective what is meant by Buddhist doctrine in the so-called “absence of ego” or “Nonself” (Shiah, 2016). Specifically, Epstein (1988) focused on that “abstract self-representation” that permits to everyone to build an image of oneself like an agent, independent, and immanent, based on a narcissistic representation of ideal Ego. “*Exposure of these representations through the non-judgmental light of mindfulness permits a simultaneous dis-identification from and integration of self-images that have often been unquestioned assumptions or split off rejections”* (Epstein, 1988, p. 65). It means that one, through meditation practice, could become aware of all the features that he abstractly ascribes to himself, but with a detachment from these images. The process of detachment from these features is represented by the “Self-transcending path” of Buddhist's tradition that aims at bringing awareness of the transitory nature of the Self, which is a feature scarcely considered in modern MBIs (Chiesa and Malinowski, 2011). By focusing on a deep introspective analysis of the Self, psychodynamic approach could be the eligible way to achieve the awareness of Self impermanence. This process is made possible by some alteration in functional parts of Ego due to the meditation practice. Indeed, this awareness can be referred to a function that implies an auto-observational capacity of Ego to be aware of itself and of what it is experiencing (Rapgay and Bystrisky, 2009). Since self-representation as agent loses its centrality along the practice, a boost of this Ego function permits the “*integration of the experience of disintegration*” of the Self (Epstein, 1988, p. 67).

At the same time meditation could further a reduction of other Ego functions. Brown and Engler's studies (as cited in Epstein, 1990) showed that experienced meditators could avoid to use defense mechanisms dealing with their internal conflicts. Meditation practice promotes a reduction of defensive function of Ego, “c*ensoring of any kind is discouraged”* (Epstein, 1990, p. 21).

In this vein, when Buddhist psychology talks about “absence of Ego,” “egolessness” (Epstein, 1988), or “emptiness,” it does not refer to a simple “nothing,” as in a lack of an Ego structure, but it involves a shift from a representation of an independent and solid Self to a conception of Self as relative, contingent and contextual (Epstein, 2015). Self is not meant as something that existed and then removed, but as something that has never been in the way we usually think about it (Epstein, 1990). “*To understand selflessness one had to first find the self as it exists, and then examine the feeling closely*” (Epstein, 2015, p. 22).

Meditation could be introduced in psychodynamic interventions, as Germer et al. (2013) proposed different ways to included mindfulness practices in psychotherapy: (a) “meditation-based psychodynamic psychotherapy,” in which TM is meant as the core of the treatment to achieve therapy purposes; b) “meditation-informed psychodynamic psychotherapy,” in which the therapist refer to Buddhist psychology without an explicit use of meditation practices during the treatment; (c) “psychodynamic therapist who practices meditation,” in which the therapist employs meditation that could lead to a development of positive qualities like empathy and compassion, and an improvement in clinical skills, like emotional regulation and management of transferal and counter transferal dynamics.

## Conclusions and future directions

The purpose of this article is to consolidate the inter-theoretical bridge between psychodynamic approach and TM, beyond the apparent incompatibility.

Our impression is that even if some authors have already worked in order to fill the gap between TM and psychodynamic psychotherapy at theoretical level, this integration could be underrated and these efforts remain isolated. This could be due mainly to ambiguities in the translation of those terms with respect to the fundaments of core concepts of both perspectives, and a lack of empirical research on psychodynamic and meditation. Psychodynamic approach could embrace those aspects of TM that have been less developed in MBIs' theory and practice. Moreover, an integration of modern mindfulness practices into a psychodynamic framework should be explored. Further empirical studies and theoretical considerations are needed to corroborate testable hypotheses and comparing classical and combined models, in order to promote the integration between these perspectives, both for clinical practice and scientific research.

## Author contributions

All authors listed, have made substantial, direct and intellectual contribution to the work, and approved it for publication.

### Conflict of interest statement

The authors declare that the research was conducted in the absence of any commercial or financial relationships that could be construed as a potential conflict of interest.

## References

[B1] Assalian P. (2013). Psychological and interpersonal dimensions of sexual function and dysfunction. Arab. J. Urol. 11, 217–221. 10.1016/j.aju.2013.07.007 26558085 PMC4442989

[B2] Bandelow B. Reitt M. Röver C. Michaelis S. Görlich Y. Wedekind D. (2015). Efficacy of treatments for anxiety disorders: a meta-analysis. Int. Clin. Psychopharmacol. 30, 183–192. 10.1097/YIC.0000000000000078 25932596

[B3] Bishop S. R. Lau M. Shapiro S. Carlson L. Anderson N. D. Carmody J. (2004). Mindfulness: a proposed operational definition. Clin. Psychol. Sci. Pract. 11, 230–241. 10.1093/clipsy.bph077

[B4] Bohlmeijer E. Prenger R. Taal E. Cuijpers P. (2010). The effects of mindfulness-based stress reduction therapy on mental health of adults with a chronic medical disease: a meta-analysis. J. Psychosom. Res. 68, 539–544. 10.1016/j.jpsychores.2009.10.005 20488270

[B5] Chiesa A. Malinowski P. (2011). Mindfulness-based approaches: are they all the same? J. Clin. Psychol. 67, 404–424. 10.1002/jclp.20776 21254062

[B6] Dalai Lama (1986). Kindness, Clarity and Insight. New York, NY: Snow Lion Publications.

[B7] Engler J. (1984). Therapeutic aims in psychotherapy and meditation: developmental stages in the representation of self. J. Transpers. Psychol. 16, 25–61.

[B8] Epstein M. (1988). The deconstruction of the self: ego and “egolessness” in Buddhist insight meditation. J. Transpers. Psychol. 20, 61–69.

[B9] Epstein M. (1990). Psychodynamics of meditation: pitfalls on the spiritual path. J. Transpers. Psychol. 22, 17–34.

[B10] Epstein M. (2008). Psychotherapy without the Self: A Buddhist Perspective. New Haven: Yale University Press.

[B11] Epstein M. (2015). Distinction–union: a Buddhist reflection on the great embrace, in Living Moments: On the Work of Michael Eige, eds Bloch S. Daws L. (London: Karnac Books), 21–31.

[B12] Flynn A. G. (2012). Fact or faith?: on the evidence for psychotherapy for adults with intellectual disability and mental health needs. Curr. Opin. Psychiatry 25, 342–347. 10.1097/YCO.0b013e328355e196 22854415

[B13] Fonagy P. Target M. (2009). Theoretical models of psychodynamic psychotherapy, in Textbook of Psychotherapeutic Treatments, ed Gabbard G. O. (Washington, DC; London: American Psychiatric Publishing), 3–42.

[B14] Fromm E. Suzuki D. T. De Martino R. (1960). Zen Buddhism and Psychoanalysis. New York, NY: Harper & Row.

[B15] Gabbard G. O. (2014). Psychodynamic Psychiatry in Clinical Practice. Arlington, TX: American Psychiatric Pub.

[B16] Galante J. Galante I. Bekkers M. J. Gallacher J. (2014). Effect of kindness-based meditation on health and well-being: a systematic review and meta-analysis. J. Consult. Clin. Psychol. 82, 1101. 10.1037/a0037249 24979314

[B17] Galante J. Iribarren S. J. Pearce P. F. (2012). Effects of mindfulness-based cognitive therapy on mental disorders: a systematic review and meta-analysis of randomized controlled trials. J. Res. Nurs. 18, 133–155. 10.1177/1744987112466087 PMC5030069 27660642

[B18] Germer C. K. Siegel R. D. Fulton P. R. (eds.). (2013). Mindfulness and Psychotherapy. New York, NY: Guilford Press.

[B19] Goleman D. (1976). Meditation and consciousness: an asian approach to mental health. Am. J. Psychother. 30, 41–54. 1259055 10.1176/appi.psychotherapy.1976.30.1.41

[B20] Grossman P. Niemann L. Schmidt S. Walach H. (2004). Mindfulness-based stress reduction and health benefits. A meta-analysis. J. Psychosom. Res. 57, 35–43. 10.1016/S0022-3999(03)00573-7 15256293

[B21] Hayes S. C. Strosahl K. Wilson K. G. (1999). Acceptance and Commitment Therapy: An Experiential Approach to Behavior Change. New York, NY: Guilford Press.

[B22] Hofmann S. G. Sawyer A. T. Witt A. A. Oh D. (2010). The effect of mindfulness-based therapy on anxiety and depression: a meta-analytic review. J. Cons. Clin. Psychol. 78, 169. 10.1037/a0018555 20350028 PMC2848393

[B23] Hunot V. Moore T. H. M. Caldwell D. M. Furukawa T. A. Davies P. Jones H.. (2013). ‘Third wave’ cognitive and behavioural therapies versus other psychological therapies for depression. Cochrane Database Syst. Rev. 10:CD008704. 10.1002/14651858.CD008704.pub2 24142844 PMC12107518

[B24] Hwang K. K. (2011). The mandala model of self. Psychol. Stud. 56, 329–334. 10.1007/s12646-011-0110-1

[B25] Johansson R. Hesser H. Ljótsson B. Frederick R. J. Andersson G. (2012). Transdiagnostic, affect-focused, psychodynamic, guided self-help for depression and anxiety through the internet: study protocol for a randomised controlled trial. BMJ Open 2:e002167. 10.1136/bmjopen-2012-002167 23257775 PMC3533089

[B26] Jung C. G. (1939). Psychological commentaries on ‘The Tibetan Book of Great Liberation’, in Collected Works of C. G. Jung, Vol. 11/II (Transl. by Hull R. F. C.), eds Read S. H. Adler G. (London: Routledge), 475–508.

[B27] Kabat-Zinn J. (1990). Full Catastrophe Living: Using the Wisdom of Your Body and Mind to Face Stress, Pain and Illness. New York, NY: Delascorte.

[B28] Keng S. L. Smoski M. J. Robins C. J. (2011). Effects of mindfulness on psychological health: a review of empirical studies. Clin. Psychol. Rev. 31, 1041–1056. 10.1016/j.cpr.2011.04.006 21802619 PMC3679190

[B29] Linehan M. (1993). Cognitive Behavioral Treatment of Borderline Personality Disorder. New York, NY: Guilford Press.

[B30] Mace C. (2007). Mindfulness in psychotherapy: an introduction. Adv. Psychiatr. Treat 13, 147–154. 10.1192/apt.bp.106.002923

[B31] Mayo-Wilson E. Dias S. Mavranezouli I. Kew K. Clark D. M. Ades A. E.. (2014). Psychological and pharmacological interventions for social anxiety disorder in adults: a systematic review and network meta-analysis. Lancet Psychiatry 1, 368–376. 10.1016/S2215-0366(14)70329-3 26361000 PMC4287862

[B32] Naliboff B. D. Fresé M. P. Rapgay L. (2008). Mind/body psychological treatments for irritable bowel syndrome. Evid. Based Complement. Alternat. Med. 5, 41–50. 10.1093/ecam/nem046 18317547 PMC2249749

[B33] Pagnini F. Philips D. (2015). Being mindful about mindfulness. Lancet Psychiatry 2, 288–289. 10.1016/S2215-0366(15)00041-3 26360065

[B34] Pagnini F. Phillips D. Bosma C. M. Reece A. Langer E. (2015). Mindfulness, physical impairment and psychological well-being in people with amyotrophic lateral sclerosis. Psychol. Health 30, 503–517. 10.1080/08870446.2014.982652 25361013

[B35] Piet J. Hougaard E. (2011). The effect of mindfulness-based cognitive therapy for prevention of relapse in recurrent major depressive disorder: a systematic review and meta-analysis. Clin. Psychol. Rev. 31, 1032–1040. 10.1016/j.cpr.2011.05.002 21802618

[B36] Piet J. Würtzen H. Zachariae R. (2012). The effect of mindfulness-based therapy on symptoms of anxiety and depression in adult cancer patients and survivors: a systematic review and meta-analysis. J. Cons. Clin. Psychol. 80, 1007. 10.1037/a0028329 22563637

[B37] Purdy J. (2013). Chronic physical illness: a psychophysiological approach for chronic physical illness. Yale J. Biol. Med. 86, 15. 23483831 PMC3584492

[B38] Rapgay L. Bystrisky A. (2009). Classical mindfulness. Ann. N. Y. Acad. Sci. 1172, 148–162. 10.1111/j.1749-6632.2009.04405.x 19735247

[B39] Recordon N. Köhl J. (2014). [Sex therapy for sexual dysfunctions]. Rev. Med. Suisse 10, 651–653. 24734364

[B40] Rosenzweig S. Greeson J. M. Reibel D. K. Green J. S. Jasser S. A. Beasley D. (2010). Mindfulness-based stress reduction for chronic pain conditions: variation in treatment outcomes and role of home meditation practice. J. Psychosom. Res. 68, 29–36. 10.1016/j.jpsychores.2009.03.010 20004298

[B41] Rubin J. B. (1999). Close encounters of a new kind: toward an integration of psychoanalysis and Buddhism. Am. J. Psychoanal. 59, 5–24. 10.1023/A:1021436704327 10078321

[B42] Safran J. (2003). Psychoanalysis and Buddhism: an Unfolding Dialogue. Somerville, MA: Wisdom Publications.

[B43] Schussel L. Miller L. (2013). Best self visualization method with high-risk youth. J. Clin. Psychol. 69, 836–845. 10.1002/jclp.22019 23775428

[B44] Segal Z. V. Williams J. M. Teasdale J. D. (2002). Mindfulness-Based Cognitive Therapy for Depression: A New Approach to Preventing Relapse. New York, NY: Guilford Press.

[B45] Shapiro S. L. Carlson L. E. (2009). The Art and Science of Mindfulness: Integrating Mindfulness into Psychology and the Helping Professions. Washington, DC: American Psychological Association 10.1037/11885-000

[B46] Shiah Y. J. (2016). From self to nonself: the nonself theory. Front. Psychol. 7:124. 10.3389/fpsyg.2016.00124 26869984 PMC4740732

[B47] Sørensen P. Birket-Smith M. Wattar U. Buemann I. Salkovskis P. (2011). A randomized clinical trial of cognitive behavioural therapy versus short-term psychodynamic psychotherapy versus no intervention for patients with hypochondriasis. Psychol. Med. 41, 431–441. 10.1017/S0033291710000292 20380779

[B48] Stern D. B. (1997). Unformulated Experience: From Dissociation to Imagination in Psychoanalysis. Hillsdale, NJ: Analytic Press.

[B49] Thompson N. J. Patel A. H. Selwa L. M. Stoll S. C. Begley C. E. Johnson E. K.. (2015). Expanding the efficacy of Project UPLIFT: distance delivery of mindfulness-based depression prevention to people with epilepsy. J. Cons. Clin. Psychol. 83, 304. 10.1037/a0038404 25495361 PMC4380523

[B50] Twemlow S. W. (2001a). Training psychotherapists in attributes of “mind” from Zen and psychoanalytic perspectives, Part I: Core principles, emptiness, impermanence, and paradox. Am. J. Psychother., 55, 1–21. 11291186 10.1176/appi.psychotherapy.2001.55.1.1

[B51] Twemlow S. W. (2001b). Training psychotherapists in attributes of “mind” from Zen and psychoanalytic perspectives, part II: attention, here and now, nonattachment, and compassion. Am. J. Psychother. 55, 22. 11291189 10.1176/appi.psychotherapy.2001.55.1.22

[B52] van der Velden A. M. Kuyken W. Wattar U. Crane C. Pallesen K. J. Dahlgaard J.. (2015). A systematic review of mechanisms of change in mindfulness-based cognitive therapy in the treatment of recurrent major depressive disorder. Clin. Psychol. Rev. 37, 26–39. 10.1016/j.cpr.2015.02.001 25748559

[B53] van Dessel N. Den Boeft M. van der Wouden J. C. Kleinstäuber M. Leone S. S. Terluin B. (2015). Non-pharmacological interventions for somatoform disorders and medically unexplained physical symptoms (MUPS) in adults, a Cochrane systematic review. J. Psychosom. Res. 78, 628 10.1016/j.jpsychores.2015.03.132 PMC10984143 25362239

[B54] Žvelc G. Černetič M. Košak M. (2011). Mindfulness-based transactional analysis. Trans. Anal. J. 41, 241–254. 10.1177/036215371104100306

